# Supplementary material for: Exploring the prokaryote-eukaryote interplay in microbial mats from an Andean athalassohaline wetland
Source: Microbiol Spectr. 2024 Mar 8;12(4):e00072-24. doi: 10.1128/spectrum.00072-24 (PMC10986560; doi:10.1128/spectrum.00072-24)
Supplement: Supplemental material — Tables S1, S2, S5, and S6 and supplemental figures. [file spectrum.00072-24-s0001.pdf]

Table S1. Location and date of sampling and physicochemical parameters measured in the water layer of the sampling poin

Site	Coordinates	Date	Temp (°C)	pH	Cond (µS/cm)	TDS (mg/l)	Salinity PSU	O_2 (%)
H6-1	20° 19' 53.1'' N<br>68° 50' 18.6'' W	07.12.2019	21.9	8.82	1464	1464	0.7	107.3
H6-2	20°20'1.5'' N<br>68°50'17.8'' W	07.12.2019	24.7	8.57	2350	2330	1.2	212
H6-3	20°20'3.9'' N<br>68°50'18.4'' W	07.12.2019	18.5	7.05	2060	2270	1.1	42.3
H6-4	20°20'7.1'' N<br>68°50'19.2'' W	07.12.2019	18	9.23	1039	829	0.5	150
H4-1	20°17'29.1'' N<br>68°53'18.3'' W	08.12.2019	5.3	8.94	31400	31400	18.9	2.6
H4-2	20°17'28.9'' N<br>68°53'18.9'' W	08.12.2019	6.5	8.79	1958	1984	0.9	0
H4-3	20°17'29.6'' N<br>68°53'23.5'' W	08.12.2019	10.9	8.37	2020	2000	1	2.2
H4-4	20°17'29.6'' N<br>68°53'23.5'' W	08.12.2019	12.9	8.6	2420	2360	1.2	36.5
H3-1	20°17'0.4'' N<br>68°53'19.5'' W	08.12.2019	17.1	8.08	570	577	0.2	113.4
H3-3	20°17'0.4'' N<br>68°53'19.5'' W	08.12.2019	22.1	8.92	1242	1120	0.6	75.5
H0-1	20°15'51.4'' N<br>68°52'27.9'' W	08.12.2019	20.9	8.92	588	588	0.2	192.2

Table S2. Samples analyzed, sequence statistics and diversity indexes. ASV, amplicon sequence variant. Sediment always refers to sediment layers below the corresponding microbial mats

Sample name	Site	Nature	16S rRNA amplicon sequences		18S rRNA amplicon sequences		Number of ASVs			Diversity indexes (prokaryotes)					Diversity indexes (eukaryotes)				
			Total	Retained high-quality	Total	Retained high-	Archaea	Bacteria	Eukaryotes	Simpson index	Chao1	Shannon-Wiener index	Species richnes	Pielou's index	Simpson index	Chao1	Shannon-Wiener index	Species richnes	Pielou's index
H0-1-M	H0	mat	30340	13692	42418	6879	6	264	112	0.92	270.00	5.45	270	0.67	0.90	112.00	4.84	112	0.71
H0-1-S	H0	sediment	27666	12555	24686	6580	118	582	103	0.99	700.56	8.72	700	0.92	0.91	103.43	4.67	103	0.70
H3-1-BF	H3	biofilm	6891	2703	11421	3043	0	142	76	0.97	142.00	6.06	142	0.85	0.96	112.00	5.16	76	0.83
H3-1-S	H3	sediment	13105	6420	31580	9110	43	337	116	0.99	380.00	7.83	380	0.91	0.92	116.00	4.82	116	0.70
H3-3-M	H3	mat	58888	38321	56058	12825	0	870	247	0.99	870.20	8.36	870	0.86	0.96	259.21	5.95	247	0.75
H4-1-M	H4	mat	57718	39906	58753	21351	1	423	73	0.98	424.00	6.92	424	0.79	0.91	73.00	4.19	73	0.68
H4-1-S	H4	sediment	41020	26026	57196	28986	12	698	101	0.99	710.00	8.09	710	0.85	0.93	102.00	4.54	101	0.68
H4-2-M	H4	mat	35784	18363	40619	18046	1	565	95	0.99	568.77	8.01	566	0.88	0.83	95.00	3.90	95	0.59
H4-2-ME	H4	mat	64188	41483	44406	14799	13	419	76	0.92	432.14	5.64	432	0.64	0.92	76.00	4.51	76	0.72
H4-4-M	H4	mat	43402	24926	46074	17320	13	908	127	0.99	921.00	8.44	921	0.86	0.94	127.33	4.72	127	0.68
H6-1-M	H6	mat	38878	20528	44488	11796	9	673	109	1.00	682.14	8.50	682	0.90	0.94	111.55	4.85	109	0.72
H6-1-S_0-5cm	H6	sediment	24752	11469	28568	9548	33	523	61	0.99	556.93	8.32	556	0.91	0.93	61.20	4.59	61	0.77
H6-1-S_5-10cm	H6	sediment	23915	11887	19429	7462	75	553	64	1.00	628.56	8.60	628	0.93	0.93	64.00	4.62	64	0.77
H6-2-M	H6	mat	35624	24333	51924	13619	2	559	160	0.99	561.43	7.85	561	0.86	0.96	162.33	5.41	160	0.74
H6-2-S	H6	sediment	15375	11294	35945	19849	18	708	159	1.00	726.00	9.07	726	0.95	0.99	159.00	7.10	159	0.97
H6-3-M	H6	mat	55205	33729	81143	46990	16	511	108	0.98	527.19	6.71	527	0.74	0.81	108.75	3.37	108	0.50
H6-3-S	H6	sediment	42470	28448	57018	25628	16	952	303	0.99	968.81	8.80	968	0.89	0.98	308.63	6.83	303	0.83
H6-3-W-CT	H6	water	57470	43121	62163	28471	227	1494	144	0.99	1725.69	9.21	1721	0.86	0.90	145.20	4.80	144	0.67
H6-4-M	H6	mat	49348	24199	69158	31579	0	215	51	0.88	215.00	4.33	215	0.56	0.87	51.00	3.39	51	0.60

Table S3. Frequency and phylogenetic affiliation of prokaryotic ASVs in Salar de Huasco samples.

Table S4. Frequency and phylogenetic affiliation of eukaryotic ASVs in Salar de Huasco samples.

These tables are very large and can be downloaded as an excel file.

Table S5. Parameters of co-occurrence network topologies

General network	Nodes	395
	Edges	1832
	Postive	1368
	Negative	464
Filtered network	Nodes	119
	Prokaryotes	58.8%
	Eukaryotes	41.2%
	Edges	104
	Diameter	2.1
	Mean distance	0.68
	Cluster	19
	Modularity	0.92

Table S6. Statistics of major clusters of co-occurrent prokaryotic and eukaryotic ASVs.

Cluster	Domain	Phylum	Class	Order	Family	Genus	Connections	Label	Presence in mat samples (%)	Presence in sediment samples (%)
Cluster 1										
dc318e4f93e5c7cf6166ad063ce2ce89	Eukaryota	Ochrophyta	Bacillariophyta	Bacillariophyta_X	Raphid-pennate	NA_Raphid-pennate	1	7	50.1	49.9
2bcdc0b08623c436bcbeca57eeee6612	Eukaryota	Ochrophyta	Bacillariophyta	Bacillariophyta_X	Raphid-pennate	NA_Raphid-pennate	1	7	50.3	49.7
81a1a52c4affb568a520c03348f8cdef	Prokaryote	Proteobacteria	Gammaproteobacteria	Ectothiorhodospirales	Ectothiorhodospiraceae	Ectothiorhodospira	3	16	58.3	41.7
79f77063b4dc797b3bd3ed0b58a21491	Prokaryote	Proteobacteria	Alphaproteobacteria	Rhodobacterales	Rhodobacteraceae	NA_Rhodobacteraceae	1	16	68.2	31.8
766f615ebb91e5ab291122da9ab3a12f	Prokaryote	Desulfobacterota_I	Desulfovibrionia	Desulfovibrionales	Desulfomicrobiaceae	Desulfomicrobium	2	13	74.4	25.6
Cluster 2										
e073b562eafd267fe500fb68805c0c1b	Prokaryote	Gemmatimonadota	Gemmatimonadetes	Gemmatimonadales	Gemmatimonadaceae	Gemmatimonas	1	15	59.9	40.1
38cd76508fe018b57eb5f757d3c24e2c	Prokaryote	Bacteroidota	Bacteroidia	Bacteroidales	Lentimicrobiaceae	Lentimicrobium	1	9	24.0	76.0
7a5c9e45b3051c4653b7c38614f692ac	Prokaryote	Bacteroidota	Bacteroidia	Cytophagales	Cyclobacteriaceae	Algoriphagus	3	9	68.6	31.4
3eb9845cd243bc2cfcdbed443da59d4f	Prokaryote	Bacteroidota	Bacteroidia	Bacteroidales	VadinHA17	SR-FBR-E99	2	9	0.0	100.0
0961323a97c36376c64a18274d521411	Eukaryota	Ochrophyta	Bacillariophyta	Bacillariophyta_X	Araphid-pennate	NA_Araphid-pennate	2	7	41.3	58.7
ed9e188eff960b30e1ac074ca08370bb	Prokaryote	Proteobacteria	Alphaproteobacteria	Sphingomonadales	Sphingomonadaceae	Sphingomonas	1	16	81.8	18.2
1acd1194f10b63af68420330712ea31f	Prokaryote	Proteobacteria	Alphaproteobacteria	Sphingomonadales	Sphingomonadaceae	Sphingomonas	4	16	81.8	18.2
e300ddf14f7cfd072b21591b4780924e	Eukaryota	Conosa	Variosea	Variosea_X	Schizoplasmodiids	Ceratiomyxella	1	4	80.5	19.5
Cluster 3										
4b96b78bdad132c42872b483e056f04e	Eukaryota	Ochrophyta	Chrysophyceae	Chrysophyceae_X	Chrysophyceae_Cluster-D	Chrysophyceae_Cluster-D_X	2	7	100.0	0.0
13cc45e417c97cc67ba8761f6a7f95c9	Prokaryote	Cyanobacteria	Cyanobacteriia	Elainellales	Elainellaceae	NA_Elainellaceae	1	11	89.6	10.4
99a00fa53f83a5613cf8df8f96056548	Eukaryota	Ochrophyta	Bacillariophyta	Bacillariophyta_X	Raphid-pennate	NA_Raphid-pennate	4	7	100.0	0.0
cb36756419ecb275b1d0d4a1e8d06bdd	Eukaryota	Ochrophyta	Bacillariophyta	Bacillariophyta_X	Raphid-pennate	Nitzschia	3	7	84.1	15.9
4205e76064917ac2c2c46f16652142c0	Eukaryota	Ochrophyta	Bacillariophyta	Bacillariophyta_X	Raphid-pennate	Nitzschia	4	7	100.0	0.0
ccfe8681b9a8b0b0fd93443ae5299f71	Prokaryote	Bacteroidota	Bacteroidia	Flavobacteriales	Flavobacteriaceae	Flavobacterium	1	9	73.2	26.8
6e5ae7e2ddd7e7c5f97d5f239af7cd1a	Eukaryota	Ciliophora	Spirotrichea	Hypotrichia	Oxytrichidae	Oxytricha	1	3		
3a83ee6f1a20f77445081d315df96bff	Prokaryote	Cyanobacteria	Cyanobacteriia	Phormidesmiales	Phormidesmiaceae	Nodosilinea	4	11	100.0	0.0
2a2fd1e0ae3b1ea9d6d7555e8f007594	Prokaryote	Proteobacteria	Alphaproteobacteria	Sphingomonadales	Sphingomonadaceae	NA_Sphingomonadaceae	1	16	100.0	0.0
0ec983f3026acebd13724448202a7036	Prokaryote	Proteobacteria	Alphaproteobacteria	Rhodobacterales	Rhodobacteraceae	NA_Rhodobacteraceae	1	16	100.0	0.0
39bb2b1b2f7a5f543f20877040c3a0f0	Prokaryote	Delongbacteria	UBA4055	UBA4055	UBA4055	UBA4055	1	12	68.8	31.2
9b90317f485a2c4ae2c7360673d0b6bf	Prokaryote	Bacteroidota	Bacteroidia	Chitinophagales	Chitinophagaceae	Phnomibacter	3	9	100.0	0.0
Cluster 4										
5b1d2e16ff623ca86fa8eba17d767269	Eukaryota	Ochrophyta	Chrysophyceae	Chrysophyceae_X	Chrysophyceae_Cluster-B2	NA_Chrysophyceae_Cluster-B2	1	7	36.9	63.1
6337c7a4c82da992142769b354699be2	Prokaryote	Proteobacteria	Alphaproteobacteria	Sphingomonadales	Sphingomonadaceae	Erythrobacter	1	16	81.2	18.8
537eae19c81d46fe5c0643ecf65df809	Eukaryota	Chlorophyta	Chlorophyceae	Chlamydomonadales	Chlamydomonadales_X	Chlamydomonas	1	2	51.0	49.0
2fae73ba8f647d54aa8087c06681299b	Eukaryota	Ochrophyta	Bacillariophyta	Bacillariophyta_X	Raphid-pennate	Raphid-pennate_X	3	7	100.0	0.0
3f784ab9fd75b6558b09b4f994af3c0b	Prokaryote	Proteobacteria	Alphaproteobacteria	Sphingomonadales	Sphingomonadaceae	Chakrabartia	3	16	70.6	29.4
bb2d2f5cced962f603ac2f00e8726bfa	Prokaryote	Gemmatimonadota	Gemmatimonadetes	Gemmatimonadales	Gemmatimonadaceae	Gemmatimonas	2	15	100.0	0.0
Cluster 5										
bcc1107831e3bccc7dadc6ad276481ea	Eukaryota	Ochrophyta	Bacillariophyta	Bacillariophyta_X	Raphid-pennate	NA_Raphid-pennate	3	7	100.0	0.0
bb1059351a482907ba7f6c8c10f24a5b	Prokaryote	Spirochaetota	Spirochaetia	Treponematales	Termitinemataceae	Treponema_G	1	17	100.0	0.0
ed306b4f6286d6042a3263e27f653d11	Eukaryota	Ochrophyta	Bacillariophyta	Bacillariophyta_X	Raphid-pennate	Raphid-pennate_X	5	7	100.0	0.0
ba50bb8de30ea52954ab7184b4fd6f92	Eukaryota	Ochrophyta	Bacillariophyta	Bacillariophyta_X	Raphid-pennate	Navicula	1	7	100.0	0.0
e7b3da6ef445a110ee3a03fc9f81ce7a	Prokaryote	Bacteroidota	Bacteroidia	Bacteroidales	VadinHA17	NA_VadinHA17	2	9	100.0	0.0
198580222aca1797ea3b6aa396b170ab	Prokaryote	Proteobacteria	Gammaproteobacteria	Burkholderiales	Burkholderiaceae	NA_Burkholderiaceae	1	16	78.1	21.9
5d121d7ec84a162e1c07a5c341695148	Eukaryota	Cercozoa	Endomyxa	Vampyrellida	NA_Vampyrellida	NA_Vampyrellida	1	1	85.6	14.4
45a51a823bf42e79bd642f159cbc3370	Prokaryote	Proteobacteria	Alphaproteobacteria	Sphingomonadales	Sphingomonadaceae	Erythrobacter	1	16	69.6	30.4
f9ec4d3cac90588134b16a966e803a14	Prokaryote	Bacteroidota	Bacteroidia	Cytophagales	Cyclobacteriaceae	JAAUQL01	2	9	67.7	32.3
034d421d0a95de4046e4eb0214e5e9a9	Prokaryote	Cyanobacteria	Cyanobacteriia	Cyanobacteriales	NA_Cyanobacteriales	NA_Cyanobacteriales	3	11	100.0	0.0
Cluster 6										
9bab90e5604ee2eaab627bcadae5cb6f	Eukaryota	Ochrophyta	Bacillariophyta	Bacillariophyta_X	Araphid-pennate	NA_Araphid-pennate	1	7	51.4	48.6
633db22e72f80415804de79aa3389624	Prokaryote	Cyanobacteria	Cyanobacteriia	Pseudanabaenales	seudanabaenaceae	Pseudanabaena	1	11	100.0	0.0
c094c0c315fcc34f4f6d3bb71590d864	Eukaryota	Cercozoa	Endomyxa	Vampyrellida	NA_Vampyrellida	NA_Vampyrellida	2	1	100.0	0.0
a42acbf774d54c48c3a415db9f0df57f	Prokaryote	Proteobacteria	Gammaproteobacteria	Burkholderiales	Burkholderiaceae	Paucibacter_A	3	16	100.0	0.0
b854c09d7116c9f0bfc5a65a973045c9	Eukaryota	Ochrophyta	Bacillariophyta	Bacillariophyta_X	Araphid-pennate	NA_Araphid-pennate	1	7	100.0	0.0
Cluster 7										
0ac506096f3f5c210c5c38e4f8bf368d	Eukaryota	Ochrophyta	Bacillariophyta	Bacillariophyta_X	Raphid-pennate	Rhopalodia	1	7	100.0	0.0
92a88ba031df04cfd8211df246df4949	Eukaryota	Ochrophyta	Bacillariophyta	Bacillariophyta_X	Raphid-pennate	Rhopalodia	2	7	100.0	0.0
c458cc5b5c5c876b94df04ce9bbd1d40	Prokaryote	Bacteroidota	Bacteroidia	Chitinophagales	Saprospiraceae	NA_Saprospiraceae	1	9	100.0	0.0
db16ac2b93eb226a1ee4d137de1fa775	Prokaryote	Proteobacteria	Gammaproteobacteria	Pseudomonadales	Halieaceae	Chromatocurvus	2	16	100.0	0.0
Cluster 8										
8f8e9b33207e42d5ece133221c5c0243	Eukaryota	Ochrophyta	Bacillariophyta	Bacillariophyta_X	Raphid-pennate	NA	1	7	0.0	100.0
c2256795315c4da2c33510434c4fb78e	Prokaryote	Cyanobacteria	Cyanobacteriia	Cyanobacteriales	NA	NA	1	11	8.8	91.3
a8a2097de09e2fb9bc806a8a0136cfec	Eukaryota	Ochrophyta	Bacillariophyta	Bacillariophyta_X	Raphid-pennate	Raphid-pennate_X	2	7	11.8	88.2
b1038a3212d3975a5a55e0a7331e7112	Prokaryote	Proteobacteria	Gammaproteobacteria	Burkholderiales	SG8-39	SCGC-AG-212-J23	3	16	0.0	100.0
aa75061450f48bf5278a56a18d87536c	Eukaryota	Ochrophyta	Bacillariophyta	Bacillariophyta_X	Raphid-pennate	Raphid-pennate_X	1	7	0.0	100.0
Cluster 9										
9bb07d58b4a3da084df1a538ef75b91c	Eukaryota	Ochrophyta	Bacillariophyta	Bacillariophyta_X	Raphid-pennate	Pseudo-nitzschia	1	7	52.4	47.6
99d54032e7b70bb0ccb5897ff95f78f8	Prokaryote	Chloroflexota	Anaerolineae	Anaerolineales	EnvOPS12	NA_EnvOPS12	1	10	34.0	66.0
edff657783390e2148c4c5290f60cb87	Eukaryota	Ochrophyta	Bacillariophyta	Bacillariophyta_X	Raphid-pennate	NA_Raphid-pennate	2	7	41.9	58.1
623109c69c8e19c5c858e8f37f21b282	Prokaryote	Proteobacteria	Gammaproteobacteria	Halothiobacillales	Halothiobacillaceae	Guyparkeria	2	16	45.1	54.9
Cluster 10										
68f6d8297618f721959e1b947ff9f493	Prokaryote	Bacteroidota	Ignavibacteria	Ignavibacteriales	Ignavibacteriaceae	IGN2	1	9	21.5	78.5
29cb722754737b032bafc5794ebd109c	Eukaryota	Ochrophyta	Bacillariophyta	Bacillariophyta_X	Araphid-pennate	NA_Araphid-pennate	2	7	34.0	66.0
c05bfbfbebd2f20c9bb0c15c43ebf470	Eukaryota	Ochrophyta	Bacillariophyta	Bacillariophyta_X	Araphid-pennate	NA_Araphid-pennate	1	7	33.3	66.7
c015ffcfa7cf8c1d0703d29df0e586ca	Prokaryote	Proteobacteria	Gammaproteobacteria	Chromatiales	Chromatiaceae	Thiocapsa	2	16	40.6	59.4
Cluster 11										
59a199c17f7338f3cc61f94c3a5edced	Eukaryota	Fungi	Cryptomycota	Cryptomycotina	Cryptomycotina_X	Cryptomycotina_XX	2	5	62.6	37.4
eab1706e57a19a653991bd7bc4973f53	Prokaryote	Proteobacteria	Alphaproteobacteria	Rhodobacterales	Rhodobacteraceae	EhC02	1	16	62.5	37.5
f79e0a19dd104445744f27b95585639b	Prokaryote	Bacteroidota	Bacteroidia	Bacteroidales	Prolixibacteraceae	Draconibacterium	1	9	0.0	100.0
4fbbdf1733d05a2fbad89a5fd7a924ce	Prokaryote	Proteobacteria	Gammaproteobacteria	Burkholderiales	Burkholderiaceae	NA_Burkholderiaceae	1	16	71.2	28.8
6362dd6ffaf6c487705e4b4c2ddebb81	Prokaryote	Cyanobacteria	Cyanobacteriia	Cyanobacteriales	Nostocaceae	Dolichospermum	2	11	74.5	25.5
4926b707b8249d90aa3aeea012bfd88a	Eukaryota	Ochrophyta	Bacillariophyta	Bacillariophyta_X	Raphid-pennate	NA_Raphid-pennate	3	7	42.2	57.8
Cluster 12										
8745767fb4dcc3282e40c3528e9aa00b	Prokaryote	Bacteroidota	Bacteroidia	Cytophagales	Cyclobacteriaceae	Algoriphagus	1	9	68.2	31.8
240efcb93a1b2639b0ad9687b5ddf211	Eukaryota	Ochrophyta	Bacillariophyta	Bacillariophyta_X	Raphid-pennate	Raphid-pennate_X	3	7	53.4	46.6
b00686e4110c3fe130eb874df763ddd3	Prokaryote	Gemmatimonadota	Gemmatimonadetes	Gemmatimonadales	Gemmatimonadaceae	JAABRT01	1	15	65.7	34.3
c13a9ef671189f49d0cc0b7d96478097	Prokaryote	Gemmatimonadota	Gemmatimonadetes	Gemmatimonadales	Gemmatimonadaceae	Gemmatimonas	1	15	63.0	37.0
Cluster 13										
d2ceb513e4e34e7c14d0e5a88aa7ca99	Prokaryote	Proteobacteria	Alphaproteobacteria	Caulobacterales	TH1-2	Aquidulcibacter	1	16	100.0	0.0
25689b3975a59e0af4337732e06869f2	Eukaryota	Ciliophora	Nassophorea	Nassophorea_X	Nassulida	NA_Nassulida	3	3	0.0	100.0
20bf68f2871a729e181e20b1a32bffa9	Prokaryote	Bacteroidota	Bacteroidia	Bacteroidales	UBA7960	SKVR01	2	9	18.1	81.9
14966980255fe5c334bc3564209cf4de	Prokaryote	Bacteroidota	Ignavibacteria	Ignavibacteriales	Ignavibacteriaceae	IGN3	1	9	29.0	71.0
1bb84f2078088527efbf501cd555230a	Prokaryote	Proteobacteria	Alphaproteobacteria	Sphingomonadales	Sphingomonadaceae	Erythrobacter	1	16	100.0	0.0
20e1edd2ef7180c06bf17d907584ca38	Prokaryote	Desulfobacterota	Desulfobacteria	Desulfobacterales	Desulfatirhabdiaceae	Desulfatirhabdium	1	13	32.6	67.4
fdc88d68afc8bbd69ba1d3b6ff6d1466	Eukaryota	Ciliophora	Spirotrichea	Hypotrichia	Oxytrichidae	Oxytricha	3	3	84.1	15.9
Cluster 14										
701383e3282f2e0868b5b8bb985e0c01	Eukaryota	Ochrophyta	Bacillariophyta	Bacillariophyta_X	Raphid-pennate	Raphid-pennate_X	3	7	71.2	28.8
2f588d3417afc605ce8162cc72694f09	Prokaryote	Proteobacteria	Gammaproteobacteria	Burkholderiales	Burkholderiaceae	NA_Burkholderiaceae	1	16	71.5	28.5
b1be65d2068e4dbd47a0394db0e57960	Prokaryote	Proteobacteria	Alphaproteobacteria	Sphingomonadales	Sphingomonadaceae	NA_Sphingomonadaceae	1	16	83.7	16.3
177e01ce2fc91e5731208079ff019355	Prokaryote	Gemmatimonadota	Gemmatimonadetes	Gemmatimonadales	GWC2-71-9	JACDDX01	1	15	79.7	20.3
Cluster 15										
bce73e8a2f692c7d435d5e81d3d070b6	Eukaryota	Ochrophyta	Chrysophyceae	Chrysophyceae_X	Chrysophyceae_Cluster-D	Chrysophyceae_Cluster-D_X	1	7	0.0	100.0
e3f806f6ebc7442f7745e1aedcadedb6	Eukaryota	Fungi	Cryptomycota	Cryptomycotina	Cryptomycotina_X	Cryptomycotina_XX	1	5	45.8	54.2
597107857d7e4ade0da855f7f4ac8961	Eukaryota	Ochrophyta	Bacillariophyta	Bacillariophyta_X	Raphid-pennate	NA_Raphid-pennate	3	7	45.2	54.8
0ab741bd7068f236fb1f6100bc48387b	Prokaryote	Bacteroidota	Bacteroidia	Flavobacteriales	Flavobacteriaceae	Flavobacterium	1	9	59.2	40.8
93286f405ddbd0dcc60049eaadfd51f1	Prokaryote	Firmicutes	Bacilli	Erysipelotrichales	Erysipelotrichaceae	UBA6182	3	14	26.3	73.7
ee5672026ab8c5137a082457f814b10b	Prokaryote	Bacteroidota	Bacteroidia	Cytophagales	Cyclobacteriaceae	NA_Cyclobacteriaceae	1	9	67.7	32.3
Cluster 16										
d0d1f1163ecd8c59d10ef4d6f413271e	Prokaryote	Firmicutes_F	Halanaerobiia	Halanaerobiales	Halanaerobiaceae	Halanaerobium	1	14	66.2	33.8
1da19ff2992959045782d88a9cb5f9ce	Eukaryota	Ochrophyta	Bacillariophyta	Bacillariophyta_X	Raphid-pennate	Navicula	3	7	86.6	13.4
8db5591bf11fe1b03e5b91439426eca2	Prokaryote	Proteobacteria	Alphaproteobacteria	Rhodobacterales	Rhodobacteraceae	NA_Rhodobacteraceae	1	16	79.3	20.7
d7824c14b705570877fb2c8a15f4784b	Eukaryota	Ochrophyta	Bacillariophyta	Bacillariophyta_X	Raphid-pennate	Raphid-pennate_X	1	7	40.0	60.0
20b47382ea6a116db68454aa9cc68b2f	Prokaryote	Desulfobacterota	Desulfobacteria	Desulfobacterales	Desulfobacteraceae	Desulfotignum	3	13	60.6	39.4
4303d0563dc9b60c95ebb6667ea85e23	Prokaryote	Desulfobacterota	Desulfobacteria	Desulfobacterales	Desulfobacteraceae	Desulfotignum	2	13	47.9	52.1
6623ec873cc7c30bff146583539628b7	Eukaryota	Cercozoa	Endomyxa	Vampyrellida	NA_Vampyrellida	NA_Vampyrellida	3	1	58.2	41.8
ecee79c5f0c1973beffc33ba2dcc8cba	Prokaryote	Proteobacteria	Gammaproteobacteria	Ectothiorhodospirales	Thioalkalivibrionaceae	Thioalkalivibrio_B	1	16	78.8	21.2
8499ea48ca5d14bb0809c5254d8b6664	Eukaryota	Ochrophyta	Bacillariophyta	Bacillariophyta_X	Raphid-pennate	Raphid-pennate_X	2	7	52.9	47.1
0c4bdb225385e3aa7450742766b59d12	Prokaryote	Desulfobacterota	Desulfobacteria	Desulfobacterales	Desulfobacteraceae	Desulfotignum	1	13	57.9	42.1
Cluster 17										
2b8ea7b003234e87949f4961abfe14c2	Eukaryota	Ochrophyta	Bacillariophyta	Bacillariophyta_X	Araphid-pennate	NA_Araphid-pennate	2	7	46.0	54.0
363b85e6107a7b76e7746caad3c6245e	Prokaryote	Bacteroidota	Bacteroidia	Bacteroidales	VadinHA17	SR-FBR-E99	1	9	26.6	73.4
2c87c68a764a0676138117fe367892ad	Eukaryota	Ochrophyta	Chrysophyceae	Chrysophyceae_X	Chrysophyceae_Cluster-C	Chrysophyceae_Cluster-C_X	1	7	57.4	42.6
cdcf16b7c5bd37921f3ca53136e8a8ee	Prokaryote	Gemmatimonadota	Gemmatimonadetes	Gemmatimonadales	Gemmatimonadaceae	Gemmatimonas	2	15	86.4	13.6
Cluster 18										
d74e0521f607b92af1021f1a1d12074d	Prokaryote	Desulfobacterota_I	Desulfovibrionia	Desulfovibrionales	Desulfomicrobiaceae	Desulfomicrobium	1	13	70.9	29.1
1e815f1b77723124c9314d1e6a312388	Prokaryote	Bacteroidota	Bacteroidia	Bacteroidales	JAAYUG01	JAAYUG01	1	9	100.0	0.0
91cf9e0333f1407780369ffa22ee0004	Eukaryota	Sagenista	Labyrinthulomycetes	Labyrinthulida	Labyrinthulaceae	Labyrinthulaceae_ANT10_3	1	8	61.7	38.3
07e2404d756f6aa57ef7cb850adaeb8b	Eukaryota	Lobosa	Tubulinea	Nolandida	Nolandellidae	NA_Nolandellidae	2	6	100.0	0.0
26594b2bbdf2d9f5c2e0428dac873bd8	Eukaryota	Conosa	Variosea	Variosea_X	Schizoplasmodiids	Ceratiomyxella	3	4	100.0	0.0
5e72d1f79a9ec7f4561fa09954fb9c2e	Eukaryota	Ciliophora	Oligohymenophorea	Scuticociliatia_2	Scuticociliatia_2_X	Cyclidium_3	4	3	100.0	0.0
e00dc8ce50f6b10adbf060a06c02696d	Prokaryote	Cyanobacteria	Cyanobacteriia	Elainellales	Elainellaceae	NA_Elainellaceae	2	11	100.0	0.0
e221d93660429da9ccbef0c2f1eaf700	Prokaryote	Cyanobacteria	Cyanobacteriia	Elainellales	Elainellaceae	RECH01	1	11	100.0	0.0
037dc1c9a772870b6c6b78f84208f235	Prokaryote	Proteobacteria	Alphaproteobacteria	Acetobacterales	Acetobacteraceae	Roseococcus	1	16	100.0	0.0
3e1536ac3d29a27281ed523d9fa239ca	Prokaryote	Bacteroidota	Rhodothermia	Balneolales	Balneolaceae	NA_Balneolaceae	1	9	100.0	0.0
2ff4a743b59b99e2886e87f77477791c	Prokaryote	Proteobacteria	Alphaproteobacteria	Rhodobacterales	Rhodobacteraceae	Yoonia	3	16	77.9	22.1
Cluster 19										
d797085c7c7eb45c99b6cb98a55e4d41	Prokaryote	Bacteroidota	Bacteroidia	Cytophagales	Cyclobacteriaceae	ELB16-189	1	9	69.3	30.7
d375de2d531e15ad51d52949a4161b99	Eukaryota	Ochrophyta	Bacillariophyta	Bacillariophyta_X	Araphid-pennate	NA_Araphid-pennate	2	7	67.3	32.7
b14adabb49c72a6cc77bb814c7b87ec5	Eukaryota	Ochrophyta	Bacillariophyta	Bacillariophyta_X	Raphid-pennate	NA_Raphid-pennate	2	7	45.9	54.1
d6283176213c91ffbabd14c929fb1ed2	Prokaryote	Desulfobacterota	Desulfobacteria	Desulfobacterales	NA_Desulfobacterales	NA_Desulfobacterales	2	13	30.0	70.0

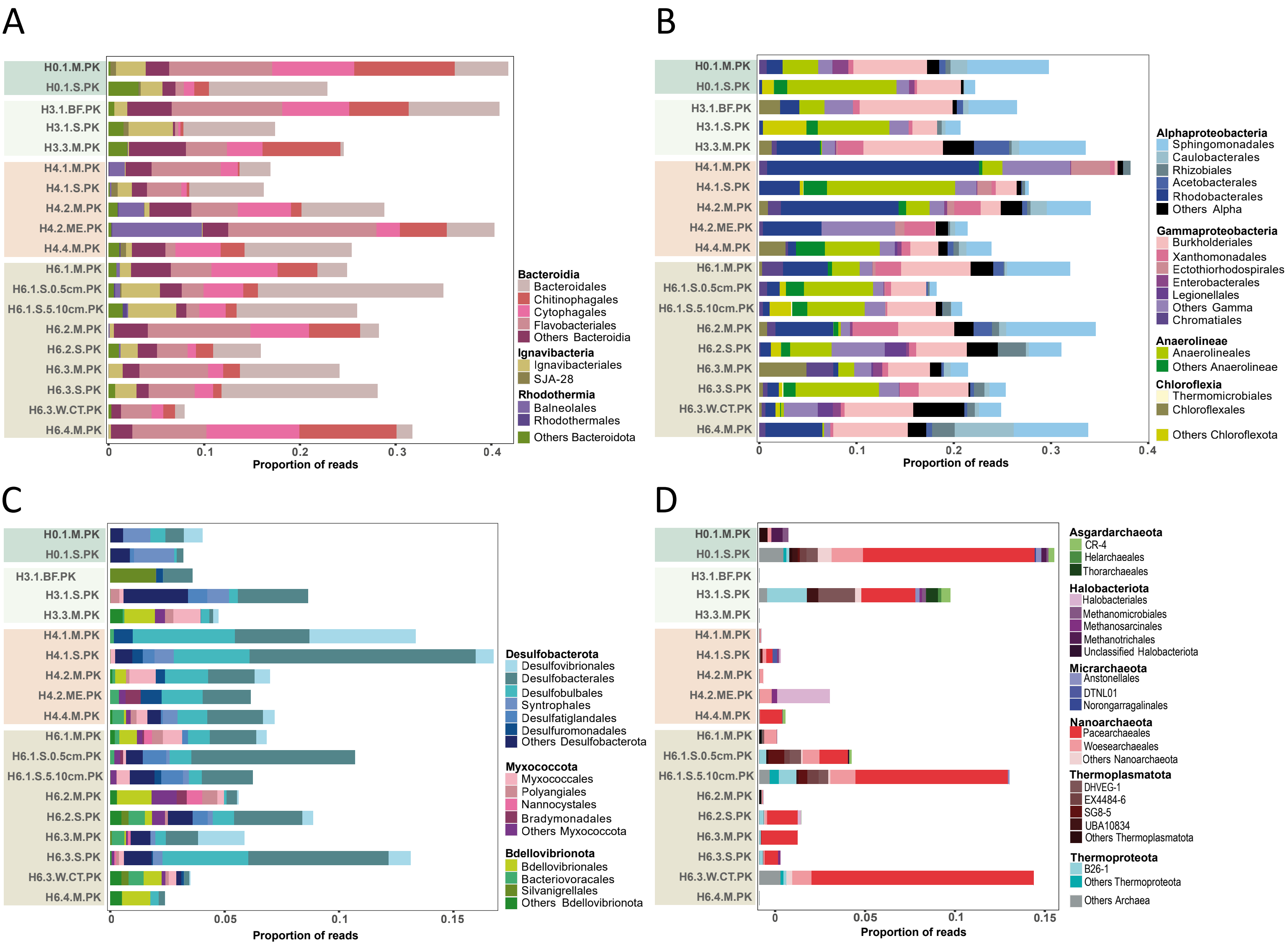

Fig. S1. Order-level taxonomic composition of selected high-rank prokaryotic taxa. A, Bacteroidetes. B, Proteobacteria (Alpha- and Gammaproteobacteria). C, Deltaproteobacteria. D, archaea.

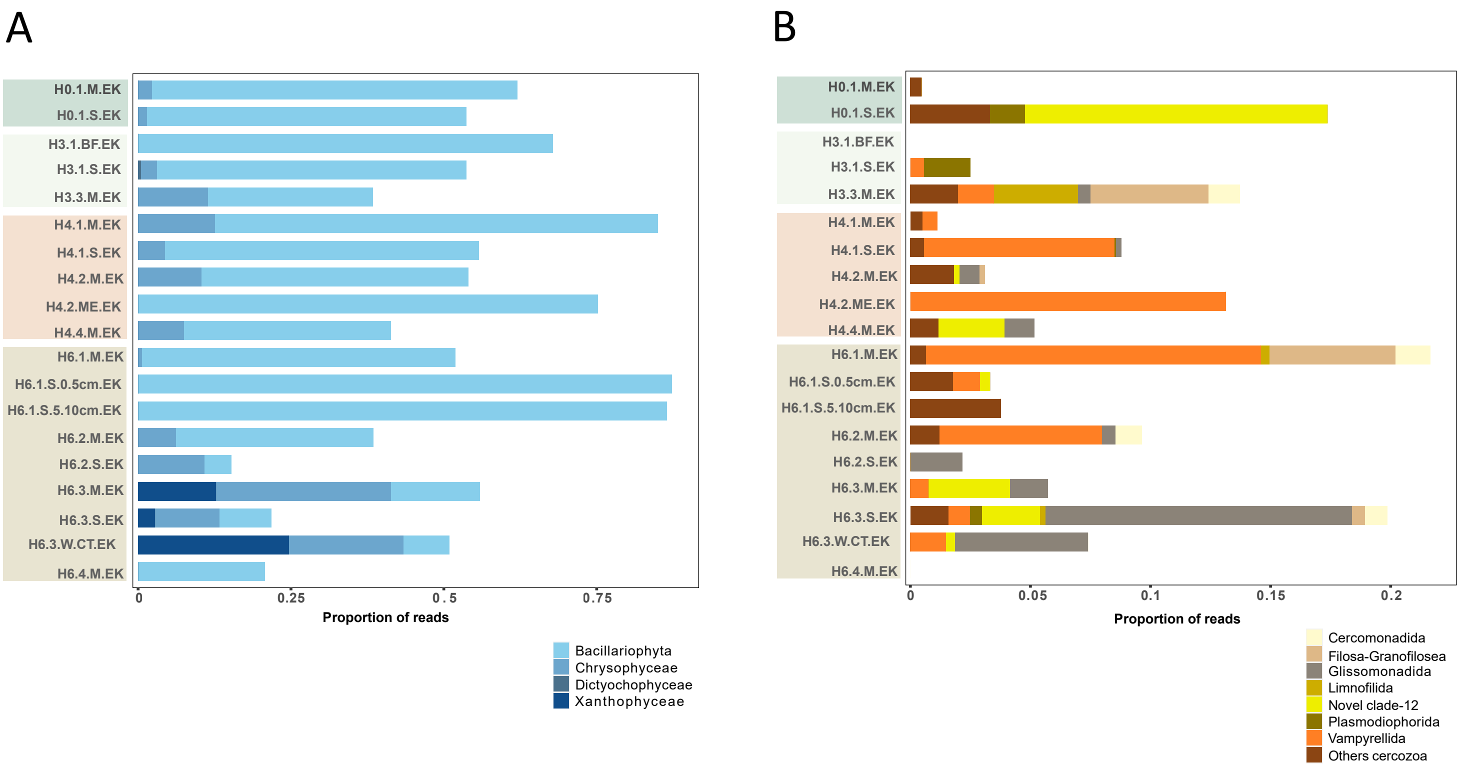

Fig. S2. Order-level taxonomic composition of selected high-rank eukaryotic taxa. A, Ochrophyta. B, Cercozoa

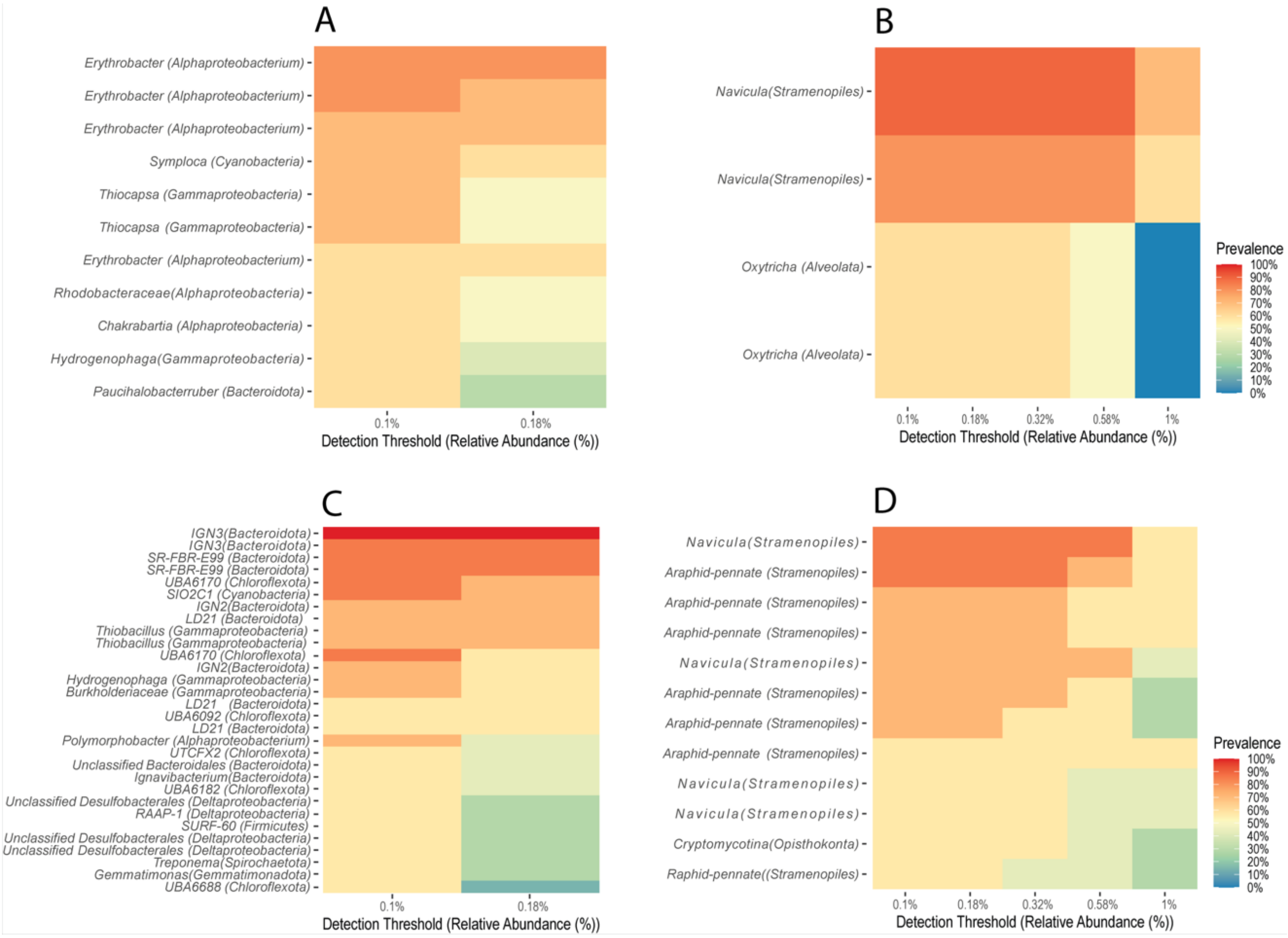

Figure S3. Composition of the prokaryotic and eukaryotic microbiomes core for microbial mats and sediment. A, prokaryotic core for microbial mats. B, eukaryotic cores for microbial mats. C, prokaryotic core for sediment samples. D, eukaryotic core for sediment samples.

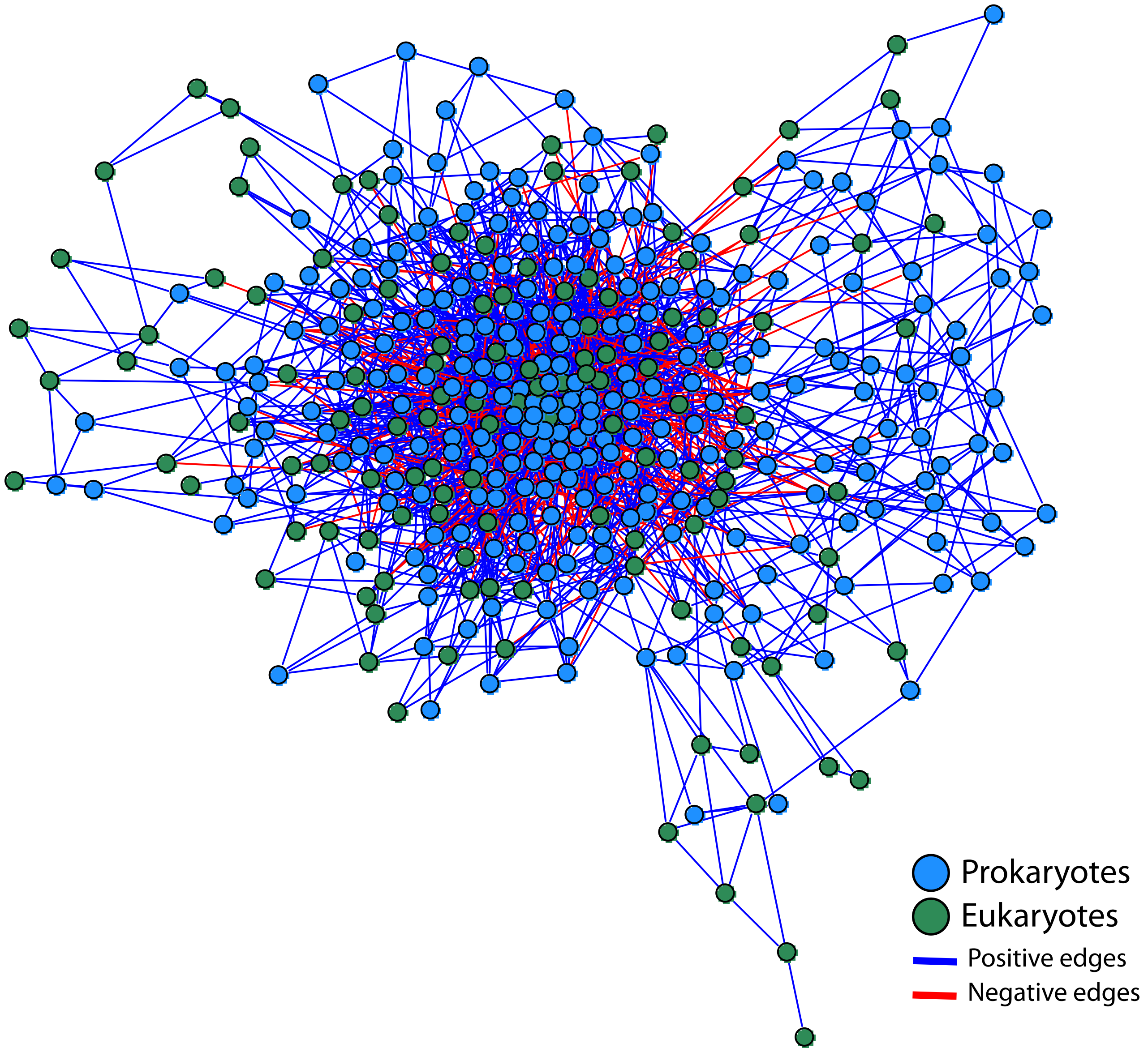

Fig. S4. Co-occurrence network of prokaryotic and eukaryotic members of mat and sediment samples from Salar de Huasco.
